# Serotoninergic and Circadian Systems: Driving Mammary Gland Development and Function

**DOI:** 10.3389/fphys.2016.00301

**Published:** 2016-07-15

**Authors:** Aridany Suárez-Trujillo, Theresa M. Casey

**Affiliations:** ^1^Animal Production and Biotechnology Group, Institute of Animal Health and Food Safety, Universidad de Las Palmas de Gran CanariaArucas, Spain; ^2^Department of Animal Sciences, Purdue UniversityWest Lafayette, IN, USA

**Keywords:** serotonin, circadian clocks, mammary gland, lactation, homeostasis, homeorhesis

## Abstract

Since lactation is one of the most metabolically demanding states in adult female mammals, beautifully complex regulatory mechanisms are in place to time lactation to begin after birth and cease when the neonate is weaned. Lactation is regulated by numerous different homeorhetic factors, all of them tightly coordinated with the demands of milk production. Emerging evidence support that among these factors are the serotonergic and circadian clock systems. Here we review the serotoninergic and circadian clock systems and their roles in the regulation of mammary gland development and lactation physiology. We conclude by presenting our hypothesis that these two systems interact to accommodate the metabolic demands of lactation and thus adaptive changes in these systems occur to maintain mammary and systemic homeostasis through the reproductive cycles of female mammals.

## Introduction

In order to keep the body in homeostasis, animals evolved multiple systems to coordinate tissue physiology. Two of these are the serotoninergic and circadian clock systems. There is growing evidence that these systems also play important roles in regulating homeorhetic adaptations to lactation, orchestrating physiological changes across the entire body around the time of birth to provide energy and nutrients to the mammary gland to support milk synthesis. Serotoninergic and circadian clock systems are both present in the central nervous system (CNS), where connections and reciprocal regulation among these systems are clear and well documented. Serotonergic factors and circadian clocks also exist in peripheral tissues, and function to regulate homeostatic processes. Recent studies support that peripheral serotoninergic and circadian systems act systemically to regulate energy mobilization during lactation, and locally within the mammary to mediate epithelial development and homeostasis. In this manuscript we review the lactation cycle and the emerging understanding of the roles of serotonergic and circadian clock systems in its regulation. We conclude by presenting our hypothesis that similar to the reciprocal regulation of these systems in the CNS, circadian and serotonergic systems interact in the mammary gland to regulate metabolic homeostasis and mediate adaptive changes needed as the animal transitions through the lactation cycle.

## Lactation cycle

In mammals lactation represents a continuation of the reproductive process, with mammary glands functioning to produce nutritional support for the offspring after birth. The lactation cycle in most placental species consists of mammogenesis, lactogenesis, colostrogenesis, galactopoiesis and involution. Mammogenesis is the growth and development of the mammary gland. Mammary development occurs primarily postnatal, and changes dynamically with reproductive state of the female (Inman et al., 2015). Completion of mammary development is only fully realized if the animal becomes pregnant. During pregnancy mammogenesis is completed and lactogenesis is initiated. Lactogenesis refers to the expression of specific genes required for the synthesis of milk by the lactocytes (mammary secretory epithelial cells) and occurs in three stages. Lactogenesis I occurs during pregnancy, whereas lactogenesis II occurs close to birth, and finally lactogenesis III commences when milk synthesis is established (Hartmann and Cregan, 2001).

Early pregnancy is marked by a high rate of epithelial cell proliferation as the ductal tree arborizes through the mammary fat pad and lobulo-alveoli form. In mid-pregnancy, lactogenesis I is initiated. In this phase, alveolar cell differentiation commences and alveoli begin to express β-casein (CSN2) and whey proteins. As parturition approaches colostrogenesis begins. Hormonal changes that occur around the time of birth initiate lactogenesis II or secretory activation, which is represented morphologically by tight junction (TJ) closure among alveolar cells (Nguyen and Neville, 1998; Stelwagen et al., 1999), and physiologically by copious secretion of milk (Pai and Horseman, 2008). Closure of tight junctions creates an impermeable barrier between blood and alveolar lumen during lactation, and results in transition from primarily paracellular transport to transcellular transport of mammary secretory product (Nguyen and Neville, 1998). Once milk secretion is established, lactogenesis III, also known as galactopoiesis, begins. Lactogenesis III is the maintenance of lactation through the homeostatic process of suckling that stimulates release of galactopoietic hormones and removal of milk from the gland. Weaning or cessation of milking stimulates the end of the lactation and initiates involution of the mammary gland. During involution, secretory tissue regresses through apoptosis of alveolar cells (Watson, 2006), and a relative increase in connective and adipose tissues as the gland returns to a less differentiated state. Another cycle of mammary development is initiated when the animal becomes newly pregnant.

### Hormonal regulation of mammary development and lactation

Peri-pubertal mammary growth is induced with the initiation of ovarian activity. In particular, initiation of ductal expansion is coincident with initiation of ovarian secretion of estradiol and progesterone (Yart et al., 2014). Once ductal tree expansion is complete, mammary development remains relatively quiescent, except for cycles of mammary epithelial proliferation and regression that occur with successive phases of the estrous/menstrual cycle (Inman et al., 2015). When the animal becomes pregnant, progesterone, placental lactogens, estrogens and prolactin (Hennighausen and Robinson, 2005) drive development and construction of lobulo-alveolar structures in preparation for lactation. Around the time of birth progesterone levels drop dramatically and levels of circulating prolactin (PRL) and glucocorticoids increase, these changes in hormonal milieu stimulate final mammary epithelial cell (MEC) differentiation to initiate lactogenesis II (Stelwagen et al., 1999; Nguyen et al., 2001). Prolactin regulates milk synthesis by binding to its cell surface receptor and activating janus kinase (JAK). JAK in turn activates signal-transducer and activator-STAT proteins 5a and 5b by phosphorylation. Activated STAT5 regulates transcription of milk proteins by binding to DNA at STAT-response elements. Glucocorticoids interact with cytoplasmic receptors which undergo allosteric change that enables the hormone-receptor complex to bind to glucocorticoid response elements, also in promoter regions of milk protein genes. Ligand bound glucocorticoid receptors can also act as a transcriptional co-activator for STAT5 and enhance STAT5-dependent transcription (Stöcklin et al., 1996). Lactogenesis III is maintained by the suckling stimulus. Suckling stimulates a neuroendocrine response that results in oxytocin, prolactin and glucocorticoid release, which function as galactopoietic hormones that maintain milk synthesis. When suckling ceases levels of galactopoietic hormones and their receptors drop and the gland regresses to a non-lactating state (Capuco et al., 2003).

### Local regulation of mammary development and lactation

Mammary development is also regulated locally through cell-cell and cell-ECM interactions. Cell-cell interactions include interactions between stromal and epithelial cells as well as between epithelial cells themselves. Stromal cells, situated around the alveoli, are often the target of systemic hormones, which stimulate production of growth factors, that in turn act in a paracrine manner to elicit effects in the adjacent epithelial structures (Anderson and Clarke, 2004). A good example of this, is estrogen regulation of ductal growth and lobulo-alveolar development during puberty and pregnancy. Stromal cells in the mammary gland express estrogen receptors (Cunha et al., 1997), and binding of estrogen to its receptor stimulates stromal cells to produce hepatocyte growth factor (HGF). HGF binds Met receptors (hepatocyte growth factor receptor) on epithelial cells, which initiates a mitogenic response that stimulates epithelial cell proliferation (Di-Cicco et al., 2015).

Cell-cell interactions among epithelial cells are important to epithelial tissue stability, communication among cells, and in the maintenance of a barrier between two milieus. As an example of the latter, at the onset of lactogenesis II, stronger junctions are formed between cells by closure of tight-junctions, which creates a barrier to separate blood constituents from the milk secretion. Tight junctions are the most apical component of cell-cell junctional complexes, and are composed of occludin proteins, and several junction-related cytoplasmic proteins, such as zona occludens-1 and 2 (ZO-1 and ZO-2). In addition to tight junctions, epithelial cells have other junctional complexes including: adherens junctions, gap junctions and desmosomes, which also mediate cell-cell communication and epithelium homeostasis. Cadherins are transmembrane proteins present in the adherens junctions. E-cadherin (CDH1) creates connections with other cadherins, but also with cytoskeletal protein (microtubules and actins) (Schneider and Kolligs, 2015). GAP junctions are intercellular channels linking the cytoplasm of adjacent cells. These channels transport small molecules such as ions, thus enabling epithelial communication and homeostasis (Stewart et al., 2015). Desmosomes (or *macula adherens*) are cell structures specialized in cell-cell adhesion and attach surface cell adhesion proteins to cytoskeletal keratin. In MECs, hormones including estrogens regulate expression of desmosomal proteins (Maynadier et al., 2012).

Differentiation of mammary epithelium is also affected by the extracellular matrix (ECM). Epithelial tissues lie on specialized ECM referred to as the basal membrane (BM). The BM is synthesized by epithelial cells, and functions to impart cell polarity (basal and luminal sides). The BM is primarily composed of collagen IV, laminins, entactin, and proteoglycans, and its composition changes dynamically with mammary development and these changes affect the activity of cells (Schedin and Keely, 2011). For example, laminin mediates the capacity of MEC to synthesize several milk proteins by cooperating with prolactin to synergistically activate STAT5 (Streuli et al., 1991, 1995; Farrelly et al., 1999).

Cell-ECM interactions are mediated, in part, by integrins, which are membrane proteins that connect extracellular and cytoplasmic milieus. Activation of integrins is integral to the regulation of branching morphogenesis of the gland and lactation phenotype in MECs (Streuli et al., 1991; Silver and Siperko, 2003). In a process referred as mechanochemical transduction, mechanical stresses induced by internal (for example growth) or external (for example gravity) forces result in conformational changes in the extracellular component and a direct stretching of the protein-cell surface. This stretching alters integrins on the cell surface and consequently activates secondary messenger pathways, and results in regulation of target genes. Stretching also causes a deformation of GAP junctions between MECs and the activation of calcium-sensitive stretch receptors which trigger secondary messenger pathways (Schedin and Keely, 2011). Activation of integrins and secondary messenger pathways in turn activate cellular metabolic activity, proliferation, differentiation and/or cell death.

In the mammary gland, the accumulation of milk that occurs with weaning stimulates signaling pathways to activate the involution process (Feng et al., 1995). Horseman and Collier (2014) proposed that locally the accumulation of milk evokes two distinct mechanisms. The first is through the accumulation of a regulatory factor in milk that binds to its cellular receptor to initiate involution; one of these factors is likely serotonin. The second is initiation of mechanochemical signal transduction pathway which is triggered through a stretch-sensing mechanism. Once these pathways are initiated, involution of the gland occurs in two phases (Lund et al., 1996). The first phase encompasses the first 48 h in rodents and is reversible. The second phase commences after those 48 h, is non-reversible and results in regression of secretory tissue. Morphologically, early involution is characterize by the detachment of epithelial cells from the alveolar structures, which is due, in part, to the tight junction break down and reduction of ZO-1 proteins. The second phase of involution, is represented by alveolar lumen area reduction and increase of inter-alveolar connective tissue (Hurley, 1987), and adipocytes (Watson, 2006).

## Systemic control of homeostasis and homeorhesis by serotonin and circadian clocks

Homeostatic processes maintain physiological equilibrium in response to, or regardless of, changes in external conditions. In contrast, homeorhesis refers to the orchestrated or coordinated changes in metabolism of body tissues necessary to support a dominant physiological state (Bauman and Currie, 1980). Homeostasis is primarily maintained by negative feedback, where as homeorhetic processes are regulated by positive feedback loops, which are characterized by their ability to maintain the direction of a stimulus and can even accelerate its effect. Pregnancy and lactation represent physiological states that homeorhetic processes are initiated. These physiological states require a huge amount of nutrients and energy to support fetal development and milk synthesis, respectively, and they challenge the equilibrium of nutrients and energy flux. Thus, pregnancy and lactation require coordinated changes in metabolism to be supported (Bell, 1995; Bell and Bauman, 1997).

Among the feedback loops that control energy homeostasis centrally is the reciprocal regulation of the serotonergic and circadian systems. In the brain, serotonin is produced in the dorsal and medial raphe nuclei (DRN and MRN), and the central-master clock is located in the suprachiasmatic nuclei of the hypothalamus. The SCN receives direct serotoninergic innervation from the raphe nuclei and also indirect through intergeniculate tract (Lovenberg et al., 1993; Prosser et al., 1993; Amir et al., 1998). In reciprocal, nervous and humoral outputs from the SCN result in circadian rhythms of serotonin levels in several brain regions including the pineal gland and raphe nuclei (Versteeg et al., 2015), with expression of the key central serotonergic gene (TPH2) in the raphe nuclei regulated in a circadian manner (Malek et al., 2007). Likewise, clock genes are expressed in serotonergic neurons (Ciarleglio et al., 2011).

The function of the circadian system is to temporally coordinate internal physiology and synchronize the organism's physiology with the environment (Froy, 2010). The central clock in the SCN acts as a master clock by temporally organizing a diverse range of physiological processes, including metabolism, across the body. Temporal input to the SCN include both photic (light) and non-photic cues, with light-dark information being the most important environmental cue for entraining the SCN. Light information is sent to the SCN via the retinohypothalamic and the geniculohypothalamic tracts (Reppert and Weaver, 2002). Serotonin is a neurotransmitter that mediates sleep, locomotor activity, and feeding behavior (Lucki, 1998), and non-photic temporal information, to include fasting/feeding and locomotor activity, is sent from serotonergic tracts to SCN.

The immediate outflow of SCN information is primarily to the medial hypothalamus, and here the SCN signal is translated into hormonal and autonomic signals for peripheral clocks located in every tissue of the body including other areas of the brain (Kalsbeek et al., 2010a,b, 2011a,b). Outputs from the SCN to the paraventricular nuclei of the hypothalamus (PVN) result in the circadian patterns of corticotropin-releasing hormone secretion. Corticotropin-releasing hormone in turn stimulates ACTH release form the pituitary which stimulates synthesis of cortisol in the adrenal gland. Circadian oscillation of plasma cortisol communicates time of day to peripheral tissues. Expression rhythms of mRNA that encodes the rate limiting enzyme of serotonin synthesis (TPH2) in dorsal and medial raphe nuclei is dependent upon daily fluctuations of glucocorticoids (Malek et al., 2007). Neurons emanating from the SCN also stimulate sympathetic neurons that innervate the pineal gland, and here melatonin is synthesized form the same precursor as serotonin, the amino acid tryptophan, according to the length of the photoperiod (Tan et al., 2014). Specifically, light acutely decreases melatonin secretion, such that melatonin secretion occurs in the dark phase of a light-dark cycle. On the contrary, serotonin is high during the daytime and low during the dark phase. Melatonin functions as a biochemical transducer of photoperiodic information to all cells in the body (Simonneaux and Ribelayga, 2003). Importantly, since melatonin is a lipophilic molecule, it can penetrate cerebral spinal fluid, and reach the dorsal raphe nuclei where it binds to melatonin receptor 1 (MT1) to influence the activity of serotonin neurons. The secretion of serotonin in the SCN by the raphe terminals as well as the diurnal serotonin rhythms in MRN and DRN change over the light-dark cycles (Versteeg et al., 2015). Thus, homeostatic information interchanged between the systems include the serotonergic system sending locomotor activity and fasting/feeding information to the SCN (van Esseveldt et al., 2000), and the SCN sending temporal information to serotonergic system.

Homeorhetic processes are also mediated through interrelated changes in central circadian and serotonergic system dynamics, with physiological changes associated with day-length being a canonical example. Animals synchronize their physiology to seasonal changes in climate and food availability by modifications in growth rate, energy balance, and reproductive capacity. Seasonal changes in photoperiod received by the SCN stimulate homeorhetic processes that adapt the animal's physiology to the changing environment. Adaptations are manifested via circadian and circannual fluctuations in the concentration and activity of a number of hormones and neurotransmitters, including serotonin. Concentration of serotonin metabolites in blood plasma is markedly lower in the winter, and the introduction of light impulses at that time of year increases the concentration of these compounds in the peripheral circulation. Therefore, the serotonergic system may be key to integrating photic and non-photic signals, such as light and food availability in the CNS by time year (Kirsz and Zieba, 2012).

## Organization of serotoninergic and circadian systems

At the cellular level distinct negative feedback mechanisms regulate circadian (transcriptional-translational feedback loop) and serotonergic (receptor-transporter competition) homeostatic processes in peripheral organs. An understanding of the manner peripheral serotonergic and circadian systems accommodate for changes in metabolic demands associated with changes in the lactation cycle are now beginning to emerge, and is reviewed below.

### Serotonin homeostatic loops

Serotonin (5-HT) is a monoamine molecule that is found in animals, plants and most unicellular organisms. Although, traditionally 5-HT is thought of as neurotransmitter that functions in the CNS to regulate mood, appetite, sleep and homeostasis, 95% of the serotonin synthesized in the body is produced in the gastrointestinal tract (Taniyama et al., 2000; Reist et al., 2003). Peripheral serotonin has systemic as well as paracrine-autocrine function, however since serotonin cannot cross the blood-brain barrier, the central and peripheral serotonergic systems are functionally separated (Namkung et al., 2015). Similar to its role in the CNS, peripheral serotonin regulates homeostatic processes, including energy homeostasis. Interestingly, while central serotonin decreases energy intake by reducing appetite and increasing energy expenditure, peripheral serotonin has seemingly an opposite role, with a net effect of energy conservation (Namkung et al., 2015).

**Figure 1:**
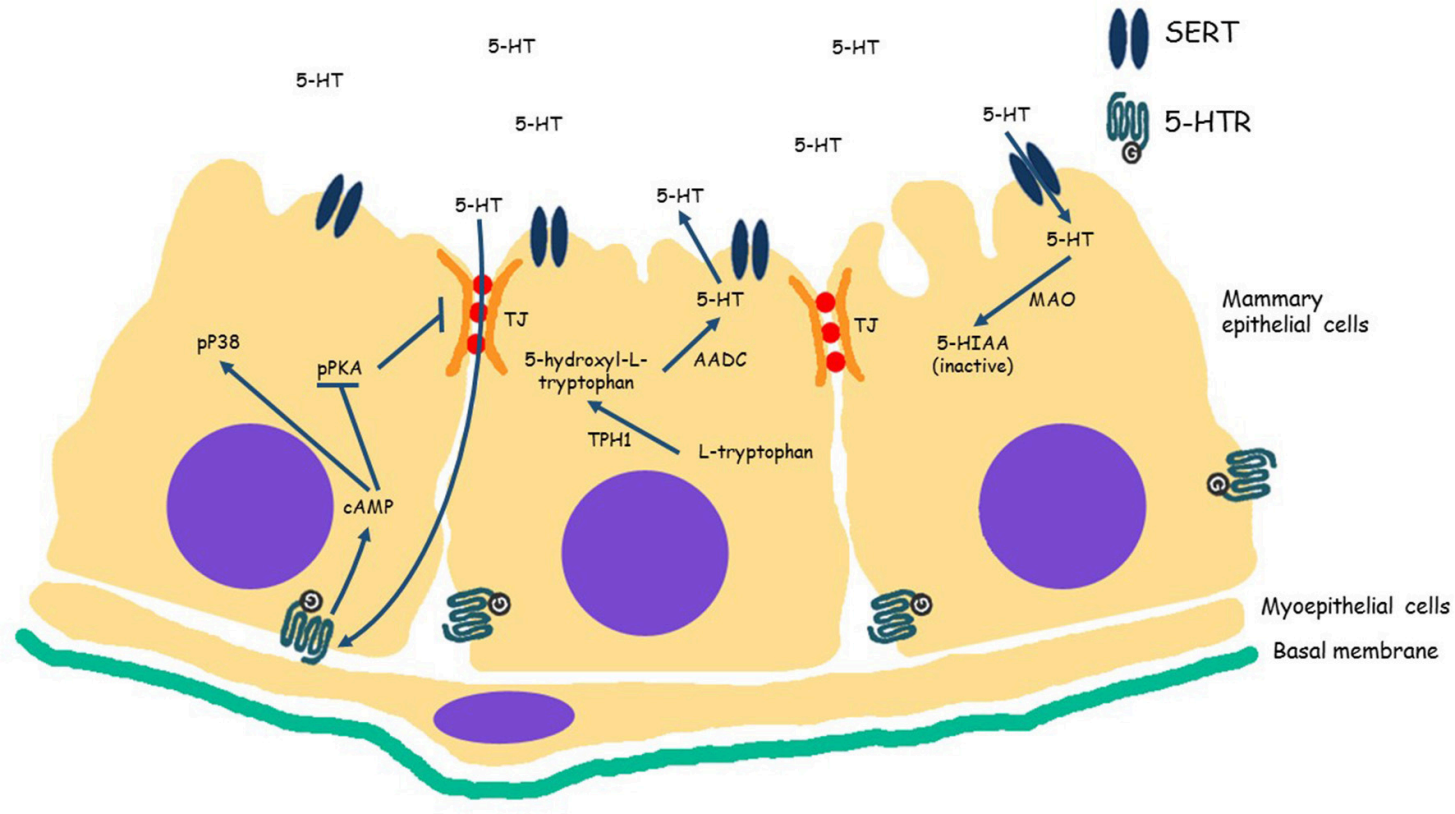
**Serotoninergic system in mammary epithelial cells**. Serotonin, 5-hydroxytryptamine (5-HT) is synthesized from L-tryptophan by the rate limiting enzyme tryptophan hydroxylase 1 (TPH1) into 5-hydroxyl-L-tryptophan. Aromatic amino acid decarboxylase (AADC) converts 5-hydroxyl-L-tryptophan into 5-HT. After synthesis, 5-HT is released in the alveolar lumen. Serotonin is either transported across cellular membrane into cytoplasm by SERT (solute carrier family 6 member 4, SLC6A4) where it is degraded by monoamine oxidase (MAO) in inactive metabolites (5-HIAA), or binds to one of its G protein coupled receptors (serotonin receptors, 5-HTR) in the cell membrane. At high levels of 5-HT, binds to receptors and increases cAMP concentration in the cell, which in turn results in decreased PKA phosphorylation (pPKA) and increased phosphorylated P38. Decrease of pPKA interferes with tight junction (TJ) integrity.

At the cellular level the serotonergic system is maintained in homeostasis through competing transporter-receptor activity (Figure 1). Serotonin is synthesized from tryptophan and is catalyzed by the rate limiting enzyme tryptophan hydroxylase 1 (TPH1) in peripheral tissues, including MECs (Matsuda et al., 2004). In the mammary gland, 5-HT is released into the alveolar lumen with other milk components during lactation. As at the neuron synapsis, the amount of 5-HT available in the alveolar lumen is controlled by a serotonin reuptake transporter (SERT), which is situated on the apical membrane of MECs (Hernandez et al., 2011). The reuptake of 5-HT into MEC by SERT, results in its degradation into inactive metabolites in the cell cytoplasm (Matsuda et al., 2004). 5-HT that is not transported by SERT can bind to cell surface receptors (5-HTR) to initiate signaling pathways that mediate its activity. Fourteen serotonin receptors (5-HTR) have been identified, and are divided into seven families (5-HTR1 to 5-HTR7) according to signaling mechanism (Roth, 1994; Reist et al., 2003). The diversity of 5-HTRs provides diverse effects of serotonin on target cells, for example in the gastrointestinal tract, stimulation of 5-HTR1 results in relaxation of smooth muscle, while binding of 5-HTR2, 5-HTR3, and 5-HTR4 results in contraction of smooth muscle.

### Peripheral serotonin in metabolic homeostasis

In the gut enterochromaffin cells secrete serotonin in response to food in the intestinal lumen, and stimulates the gut to contract around the food (Reist et al., 2003). Excess secreted serotonin is taken up by platelets in the veins draining the gut and stored. Upon platelet activation gut-derived serotonin is released and functions to control hemodynamics. Gut-derived serotonin also promotes gluconeogenesis and suppresses hepatic glucose uptake through activation of 5-HTR2B on hepatocytes, indicating that gut derived serotonin regulates hepatic glucose metabolism (Lesurtel et al., 2006). Alterations in serotonin levels and signaling also regulate bone mass (Yadav et al., 2008a,b). Mice that lack brain serotonin have low bone density, while mice that lack gut serotonin have high bone density, suggesting contradictory roles of peripheral and CNS serotonin in bone homeostasis. In humans, increased blood serotonin levels have been shown to be significant negative predictor of low bone density (Yadav et al., 2010). Serotonin can also be synthesized, albeit at very low levels, in bone cells, and its actions on bone cells is mediated by three different receptors. Through 5-HTR1B receptors, 5-HT negatively regulates bone mass, while it does so positively through 5-HTR2B receptors and 5-HTR2C receptors. Serotonin is also synthesized in adipose and mammary tissue. In white adipose tissue serotonin increases energy storage and adipogenesis in through 5-HTR2A and inhibits adaptive thermogenesis in brown adipose tissue through 5-HTR3 (Namkung et al., 2015).

Evidence for role of peripheral 5-HT as a homeorhetic regulator during pregnancy and lactation (Bell, 1995; Bell and Bauman, 1997) stems from its actions on energy mobilization and calcium metabolism. Serotonin production increases dramatically during pregnancy. *Ex vivo* mRNA expression analysis of pancreatic islet cells isolated from non-pregnant mice vs. mice at gestation day 15, revealed that TPH1 was one of the most highly induced genes in islets during pregnancy. Parallel to TPH1 mRNA and protein induction, islet serotonin content from pregnant animals increased to a peak level that was 200-fold higher than basal non-pregnant levels (Schraenen et al., 2010). Additional studies found that during pregnancy prolactin and placental lactogen induce 5-HT synthesis in pancreas, and in turn 5-HT induced upregulation of the insulin-producing β-cell mass, which is required to support the physiological demands for insulin during pregnancy (Kim et al., 2010, 2015; Ohara-Imaizumi et al., 2013).

Circulating 5-HT levels is associated with calcium trafficking during lactation. Studies in cattle showed that circulating levels of 5-HT change dynamically from pregnancy through late lactation. Serum 5-HT concentration in normal dairy cows was stable during pre-partum period, decreased during the transition from pregnancy to lactation, and then increased several days after parturition (Laporta and Hernandez, 2015; Moore et al., 2015). Studies of lactating mice with conditional knockout of TPH1 in mammary beginning in late pregnancy created by crossing WAP-Cre mice with TPH1 floxed mice, found circulating serotonin concentrations were approximately half those of wild-type animals (700–800 ng/ml TPH1 knockout vs. 1500 ng/ml (Laura Hernandez, personal communication), suggesting that mammary derived 5-HT is a primary source of systemic serotonin during lactation. In dairy cows serum 5-HT concentration was positively correlated with calcium and parathyroid-hormone related peptide (PTHrP) on the first day postpartum (Laporta et al., 2013a). Supplementation of lactating rodents with 5-hydroxy-L-tryptophan (5-HTP), which raises serum 5-HT concentration, increased serum levels of serotonin, PTHrP, and calcium, as well as milk calcium levels (Laporta et al., 2013c). In vitro studies of primary bovine MECs treated with lactogenic hormones (prolactin, insulin, and cortisol), showed that adding 5-HT to the culture media stimulated PTHrP mRNA expression (Horseman and Hernandez, 2014). Together these studies support that 5-HT stimulates mammary to express PTHrP, which travels through circulation to bone and initiates mammotropic signaling resulting in a higher osteoclastic activity and calcium resorption, thus increasing calcium concentration in serum. Studies of lactating mice with mammary specific knockout of TPH1 found lower levels of calcium transporters genes (PMCA2, CaSR, ORAI-1, SERCA-2, SPCA1, and 2) than wild type controls. Injections of TPH1 knockouts with 5-HTP resulted in partial recovery in expression levels of these calcium-related genes (Laporta et al., 2014). Supplementation of lactating rodents with 5-hydroxy-L-tryptophan (5-HTP), which raises serum 5-HT concentration, results in upregulation of key gluconeogenic, glycolytic, and energy metabolism enzymes in the liver (Laporta et al., 2013b), and increased glucose transporters 1 and 8 (GLUT 1 and 8) mRNA expression in mammary glands. Thus, these studies support a role for 5-HT in eliciting homeorhetic processes in multiple tissues from mammary to bone to liver, and at multiple levels from systemic to local to support pregnancy and lactation.

### Local control of mammary gland function by serotonin: biphasic role of serotonin in mammary gland function

Mammary 5-HT has paracrine-autocrine functions, and which are particularly important to maintaining lactational homeostasis. The rate limiting enzyme TPH1 is expressed in mice mammary tissue during different states of the mammary gland development (nulliparous, pregnancy, lactation and involution), but it is highest during the last period of pregnancy (after day 15 of pregnancy), followed by day 10 of lactation (Matsuda et al., 2004). Early studies of the role of 5-HT in mammary showed it functions as a lactation inhibitor (Matsuda et al., 2004; Stull et al., 2007; Hernandez et al., 2008, 2011). However, subsequent investigations support a broader paracrine-autocrine role of serotonin, including regulation of MEC function and morphology (Pai and Horseman, 2008; Pai et al., 2015), as well as initiation of mammotropic signaling to the bone in order to increase calcium bioavailability (Hernandez et al., 2012).

5-HT action on MEC is biphasic and concentration dependent. Pai and Horseman (2008) showed relatively low levels of 5-HT increases the expression of milk proteins mRNA and high levels of 5-HT decreases milk protein production. The result of this biphasic regulation is that the serotonergic system is maintained in homeostasis, through the combination of 5-HT synthesis and release, cellular reuptake (by SERT) and degradation, or milk removal by nursing or milking (Hernandez et al., 2011). During lactation 5-HT is kept in a low concentration in milk, and the effect of this low amount of 5-HT is promotion of milk synthesis. Furthermore, Pai et al. (2015) described that the lack of 5-HTR type 7 ends in a disruption of the mammary function and morphology. Therefore, the effect of 5-HT is not only related with milk protein synthesis, it is also associated with the epithelium integrity.

The inhibitory role of 5-HT is primarily evident when milk accumulates in gland at weaning (in nursing animals) or milking cessation (in dairy animals). Accumulation of milk creates pressure on MECs, and this mechanical signal enhances the serotonin system by stimulating increased TPH1 expression (Matsuda et al., 2004; Horseman and Collier, 2014), and consequently higher levels of 5-HT is secreted into alveolar lumen. High concentrations of 5-HT in the alveolar lumen results in disruption of tight junctions between MECs (Stull et al., 2007) and apoptosis is initiated (Hernandez et al., 2011), thus initiating the first phase of the involution process (Hernandez et al., 2008). Pai and Horseman (2008) elucidated the molecular pathway of 5-HT tight junction disruption, which proceeds through serotonin binding to the 5-HT receptor 7 (5-HTR7). Binding of serotonin to 5-HTR7 increases cAMP levels. High levels of cAMP results in decreased levels of activated PKA (phosphorylated-PKA, pPKA) and activation of P38, through phosphorylation (pP38). Loss of pPKA decreases ZO-1 and 2 proteins in the tight junctions, and pP38 promotes apoptosis. Furthermore, Matsuda et al. (2004) found long term exposure (10 days) of mammary explants from late pregnant mice to 5-HT induced a loss of mammary morphological differentiation and a high number of apoptotic bodies, compared to the positive control tissue (treated with hydrocortisone, insulin and prolactin, alone). In contrast, they demonstrated that reducing the 5-HT amount in the explants by treating them with a 5-HT receptor blocking agent (Methysergide) or a suppressor of TPH1 (PCPA, p-chlorophenylalanine), resulted maintenance of mammary differentiation.

In conclusion, peripheral serotonin has a wide spectrum of actions, but in general is an energy-saving molecule, inducing gluconeogenesis in liver, and in adipose tissue stimulating adipogenesis. During pregnancy and lactation, serotonin functions as a homeorhetic factor that results in the shuttling of energy metabolites, and nutrients (such as calcium) to the fetus and the mammary gland. Once lactation is established serotonin plays a central role in maintaining mammary epithelial homeostasis, working in a biphasic dose dependent manner.

### Circadian clocks: Input-outputs based system

**Figure 2:**
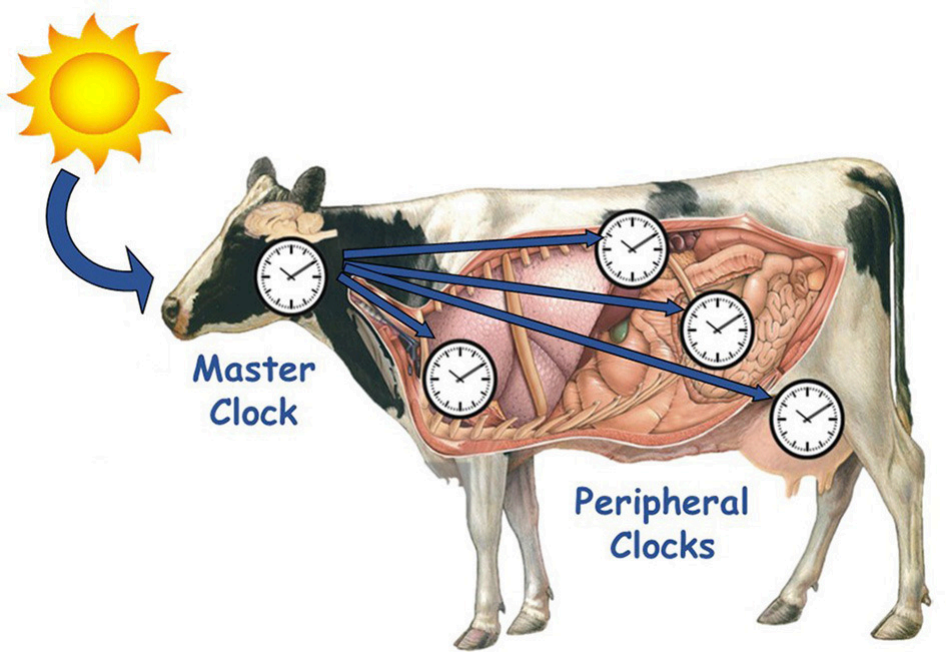
**Hierarchical organization of circadian timing system in mammals**. The master clock in the suprachiasmatic nuclei (SCN) of the hypothalamus, receives external and internal temporal information, integrates it, and then sends outputs (hormonal or neuronal cues) to the peripheral clocks that exist in every tissue of the body to coordinate internal physiology and synchronize it to the environment.

In mammals, the circadian timing system is hierarchically structured, and organized in two levels: central and peripheral clocks. The central clock, or master clock, in the SCN receives temporal information, integrates it and then sends outputs to the peripheral clocks that exist in every tissue of the body (Figure 2; Weaver, 1998; Dunlap, 1999). In turn, peripheral clocks receive these inputs of temporal information, integrate it into the molecular clock, and translate it into circadian rhythms of gene and protein expression. The specific outputs of peripheral clocks are less well defined than that of the SCN, although global temporal expression profiles of liver, adipose, mammary and heart tissues revealed that 3–10% of genes expressed in these tissues exhibited circadian patterns, and were found to be involved in rate-limiting steps critical for organ function (Akhtar et al., 2002; Panda et al., 2002; Storch et al., 2002; Ando et al., 2005; Maningat et al., 2009).

**Figure 3:**
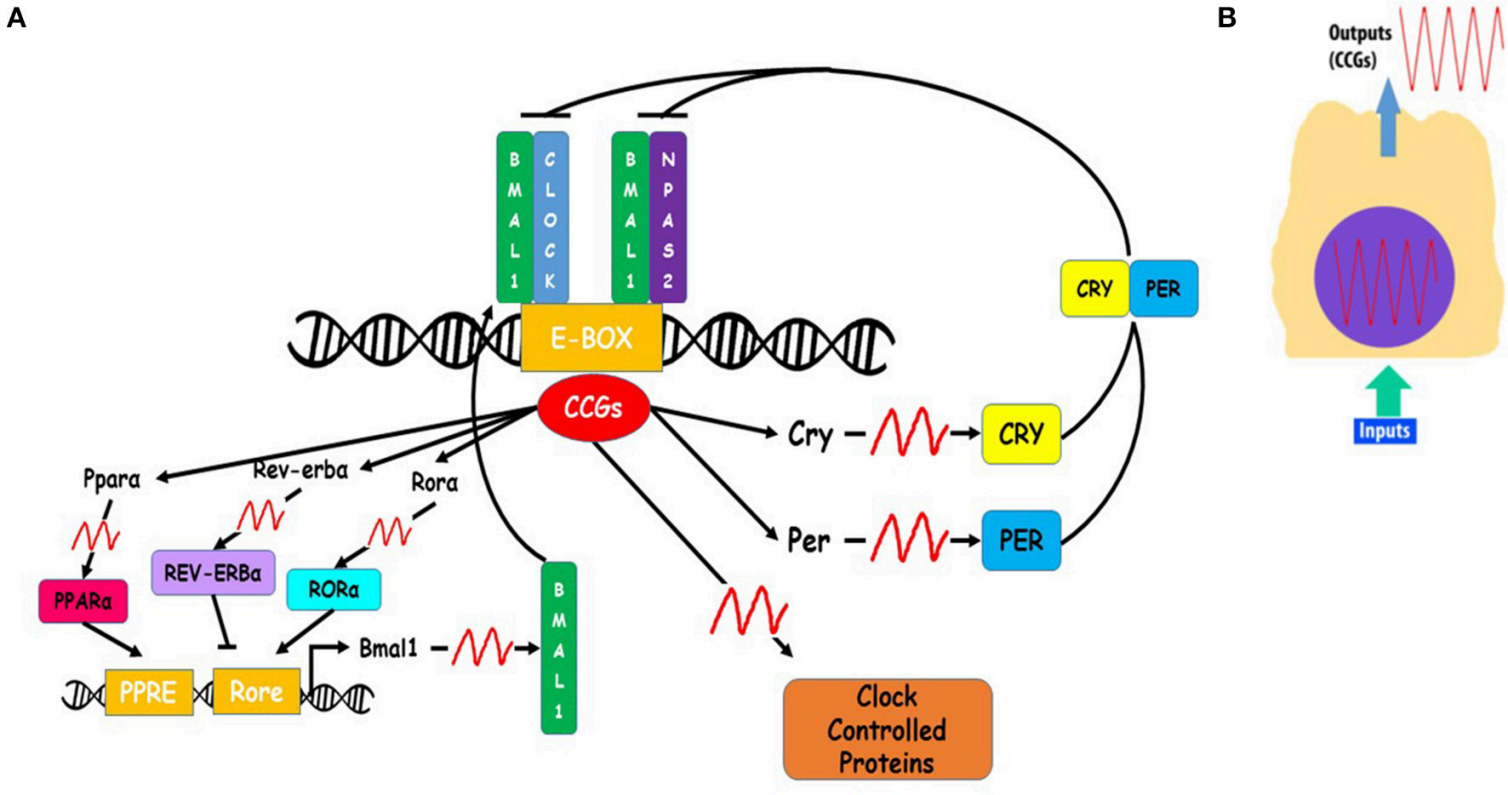
**Schema of cellular clocks' molecular mechanism**. **(A)** Circadian clock molecular mechanism is based on a transcription-translation feedback loop, in which Aryl hydrocarbon receptor nuclear translocator-like protein 1 (ARNTL, aka BMAL1), Circadian Locomotor Output Cycles Kaput (CLOCK) and Neuronal PAS Domain Protein 2 (NPAS2) constitute the positive limb. The heterodimers BMAL1-CLOCK or BMAL1-NPAS2 bind the promoter regions (E-boxes) of clock-controlled genes (CCGs). Among CCGs are Cryptochrome (Cry 1 and 2) and Period (Per 1, 2, and 3) genes, which encode for CRY 1 and 2, and PER 1, 2, and 3 factors, the negative limb of the loop. CRY and PER factors prevent BMAL1-CLOCK and BMAL1-NPAS2 binding activity in promoter regions of on CCGs. BMAL1 is also regulated by two other CCGs, REV-ERVα and RORα, which repress or activate, respectively BMAL1 expression. **(B)** Every cell has a self-sustained molecular clock that promotes CCGs oscillating expression based on a circadian rhythm. Inputs of temporal information produce changes in temporal expression of clock core genes (BMAL1, CLOCK, NPAS2, CRY, or PER), which is translated in temporal expression patterns of CCGs.

The molecular mechanism of circadian clocks is a transcription-translation feedback loop of core clock genes (Figure 3). Among circadian core clock genes are: Aryl hydrocarbon receptor nuclear translocator-like protein 1 (ARNTL, aka BMAL1), Circadian Locomotor Output Cycles Kaput (CLOCK), Neuronal PAS Domain Protein 2 (NPAS2), Period 1, 2, and 3 (PER1, PER2, PER3) and Cryptochrome 1 and 2 (CRY1, CRY2) (Lee et al., 2011). BMAL1 and CLOCK or BMAL1 and NPAS2 form the positive loop of the molecular clock. As a heterodimer (Reick et al., 2001), BMAL1-CLOCK functions as a transcription factor which binds E-Boxes promoter elements (CACGTG, canonical sequence or CANNTG, non-canonical sequences) present in clock-controlled genes (CCGs) to drive their expression (Lyons et al., 2000). Among CCGs are the core clock period (PER1, PER2, PER3) and cryptochrome (CRY1 AND CRY2) genes, which make up the negative arm of the molecular clock (Darlington et al., 1998). PER and CRY genes are translated, and their proteins accumulate in the cytoplasm where they form heterodimers that are translocated into the nucleus. In the nucleus PER-CRY prevent the promoter binding activity of BMAL1-CLOCK. The transcription-translation feedback loop occurs in a 24 h periodicity, resulting in circadian rhythms of core clock genes. BMAL1 expression is regulated by two of its transcriptional targets, nuclear receptors REV-ERBα and RORα, which repress or activate, respectively, BMAL1 transcription (Guillaumond et al., 2005).

There is reciprocal regulation among circadian timing and metabolic systems, and this is evident in the fact that circulating levels of metabolites such as glucose, fatty acids, NAD+/NADH and AMP/ATP are able to regulate clock molecular mechanism (Froy, 2012). Peroxisome proliferator-activated receptor α (PPARα) is a transcription factor that reciprocally regulates BMAL1 expression. Specifically, PPARα is a member of the nuclear receptor family that functions as a transcription factor when bound by free fatty acid and regulates expression of genes that control lipid and glucose metabolism. Expression of PPARα is mediated by the BMAL1-CLOCK heterodimer. In turn, PPARα binds to the peroxisome-proliferator response element (PPRE) to activate BMAL1 expression (Froy, 2012). Thus, the integration of the circadian and metabolic system occurs in part through the reciprocal regulation of BMAL1 and PPARα. Furthermore, the hormone ghrelin, which is synthesized in the stomach after feeding, is able to go through the brain-blood barrier and affect SCN metabolic activity (Carlini et al., 2004). In addition, timing of food intake is an input to circadian clocks in peripheral tissues (Damiola et al., 2000; Stokkan et al., 2001; Eckel-Mahan and Sassone-Corsi, 2013). Rodent studies have shown that timing of food intake is a stronger input to peripheral clocks than temporal cues on light-dark cycle, as restricting food intake to normal times of rest, shifted circadian rhythms of peripheral clocks in liver, kidney, heart, and pancreas to time of food availability, making them out phase with the clock in the SCN, which remained synchronized to the light-dark cycle (Froy, 2010).

Similar to the reciprocal regulation of the circadian and metabolic systems of the body, reproductive and circadian processes appear to be integrated and reciprocally regulated. Reproduction is orchestrated by multiple hormones that exhibit circadian rhythms of secretion. This mediation occurs in part through SCN outputs to the paraventricular nuclei of the hypothalamus (Challet, 2015). The PVN neuroendocrine neurons secrete neurotransmitters and neuropeptides that are transported through hypophyseal portal system to the pituitary gland (Buijs et al., 2003), and from there, pituitary hormonal secretion activates secondary glandular organs such as adrenal and thyroid glands and gonads. The influence of the circadian timing system on reproduction is most evident in seasonal breeders such as sheep, for which melatonin acts within the hypothalamus to mediate control of seasonal changes in gonadotrophin secretion and gonadal activity (Lincoln and Richardson, 1998; Harrison et al., 2008). In this way, seasonal animals can regulate their reproductive cycle to optimize the time of year young are born (Barrett and Bolborea, 2012).

In reproducing females (i.e., pregnant and lactating) the survival of offspring becomes a priority, and thus cues emanating from the concepts during pregnancy and the neonate during lactation likely play an important role in coordinating maternal functions. For example, Wharfe et al. (2011) demonstrated that although circadian core clock genes are expressed in rat placenta, and positive core clock genes were increased during late gestation, they did not exhibit robust circadian rhythms of expression. Investigators speculated that attenuated rhythms of clock genes' expression in placenta may ensure around-the-clock activation of downstream genes that mediate metabolic processes, vascular growth and/or substrate supply. During the transition from pregnancy to lactation changes occur in core clock genes' expression across multiple tissues (Casey et al., 2009, 2014). In particular robustness of core clock genes' expression rhythms is increased in hepatic tissue and the SCN during lactation. Amplitude of the core clock gene PER2 expression rhythm in the SCN was greater in lactating vs. pregnant mouse dams. The PER2 gene, which is one of the major transcriptional targets of CLOCK-BMAL1 complex, is involved in this central clock resetting (Nagano et al., 2009). Changes in central and hepatic clocks were speculated to be needed to compensate for the negative energy balance the dam is in at the onset of lactation. Moreover, the hypothalamus-pituitary-adrenal (HPA) axis also changes responsiveness in late pregnancy and lactation, these changes maintain relatively high basal levels of glucocorticoids with an attenuation of circadian oscillation (Brunton et al., 2008; Windle et al., 2013). Thus, similar to serotoninergic system, circadian clocks regulate metabolic homeostasis, and changes in molecular clocks and rhythms during pregnancy and lactation likely reflect adaptive changes to support these physiological states.

### Inputs and outputs in mammary gland clocks

Mammary tissue expresses circadian core clock genes, and their relationship with mammary development and lactation is beginning to be elucidated. Temporal analysis of core circadian clock genes' expression at different stages in the mouse mammary gland (virgin, pregnant, lactation and involution), showed dynamic changes in core clock genes' expression with phase of reproduction (Metz et al., 2006; Casey et al., 2014). Particularly interesting is that BMAL1 steady state mRNA levels across the circadian cycle are reduced in mammary tissue from virgin and early pregnant mice, and then increase in glands from late gestation and lactating mice. In contrast PER2 expression patterns were relatively reduced in lactating vs. pregnant glands. Furthermore, during involution, the expression of BMAL1 was reduced and PER2 increased (Metz et al., 2006). Together the studies demonstrate that mammary circadian clock follows a cycle of changes in its components along the gestation/lactation/involution cycle.

During lactation there is an attenuation of multiple core clocks genes' expression rhythms in the mammary gland, and an increase in abundance of BMAL1 and CLOCK protein levels across the entire circadian cycle (Casey et al., 2014). Circulating glucocorticoid and prolactin levels during lactation exhibit circadian rhythms that are superimposed by suckling response release, and together likely influence mammary clock dynamics. Using the HC11 cell culture model, it was found that treatment with glucocorticoids impacted core clock gene expression (Casey et al., 2014). Moreover, prolactin treatment alone significantly increased amplitude of BMAL1 expression, but had no effect on period length. In addition, prolactin induced bi-directional phase shifts in BMAL1 expression, which depended on the phase at which it was administered. Thus, these data indicated that prolactin and glucocorticoids affect mammary clock dynamics, and timing of prolactin stimulus can shift the phase of the clock. In addition, continuous exposure to prolactin and glucocorticoids in culture (vs. 2 h treatment), caused an attenuation of core circadian clocks' gene expression. Together suggesting that the attenuation of core clock gene's expression during lactation in mice is due in part to frequent suckling in early lactation, resulting in relatively continuous exposure to potent inputs to molecular clocks, glucocorticoids and prolactin. Oxytocin (OT), the other important hormone released with the initiation of pup suckling, induces milk ejection. Suckling stimulation causes release of OT from hypothalamic neurons terminating in the posterior pituitary. Upon release in circulation, OT travels to mammary and binds receptors on myoepithelial cells, stimulates cells to contract around underlying aveoli, and results in milk ejection from aveolar structure (Lollivier et al., 2006; Oguro et al., 1982). Oxytocin also travels to anterior pituitary where it binds to receptors on lactotrophs and stimulates PRL secretion (Bertram et al., 2010). Thus, OT has at least an indirect effect on mammary clock through regulation of PRL release. Timing of food intake also appears to be an input to the mammary clock during lactation. A study conducted by Ma et al. (2013) showed that restricting feeding times in rodents caused shifts in mammary clocks genes' expression and circadian variations of milk fat synthesis by affecting lipogenic regulators (SREBP1c and Spot 14) and milk fat synthetic enzymes (FASN and SCD1). Although, normally driven by the SCN, under certain physiological conditions peripheral clocks may function independently and synchronize their activity with a stimulus that is more important for physiological function of that particular tissue. These studies support that during lactation the clock in the mammary gland is responsive to suckling cues from the neonate as well as substrate availability.

Mammary gland clock function is widely unknown, but studies, including photoperiod studies, support a potential role in regulation of development as well as metabolic output, i.e., milk synthesis. Temporal transcriptome analysis revealed that 7% of the genes expressed in lactating breast exhibited circadian oscillation (Maningat et al., 2009). In addition to core circadian clocks genes, circadian oscillation genes were involved in cell development, growth, and proliferation, apoptosis, and intra-cellular signaling cascade. Work with HC11 cells, have demonstrated changes in expressions of the clock core genes between undifferentiated and differentiated states (untreated or treated with lactogen media, respectively). Similar to what is evident *in vivo* with transition from pregnancy to lactation, upon differentiation HC11 cells express a greater abundance of BMAL1 and CLOCK proteins and PER2 protein level is reduced relative to undifferentiated cultures (Casey et al., 2014). Studies from our laboratory, showed that reducing levels of CLOCK protein in HC-11 cells using shRNA (shCLOCK cells) caused higher rates of growth compared to wild-type cultures. Consistent with higher growth rate was a higher expression of cell cycle regulator cyclin D1 and lower expression of tumor protein 63 (P63) in shCLOCK transfected HC11 cells vs. wild-type controls (Casey et al., in revision). *In vivo* studies of CLOCK mutant mice support a role for the circadian system in regulation of lactation competency. *Clock*-Δ*19* mice have an ENU-induced mutation that affects transactivation properties of CLOCK, and results in disruption of behavioral rhythmicity, loss of rhythmic gene expression, and down-regulation of CLOCK-BMAL1 target genes (Antoch et al., 1997; King et al., 1997; Panda et al., 2002). Studies of circadian regulation of reproduction in the *Clock*-Δ*19* line of mice found this mutation has minimal effects on growth and development of pups during gestation, however litter growth and survival is significantly decreased postnatally (Kennaway et al., 2004; Dolatshad et al., 2006; Hoshino et al., 2006). Hoshino et al. (2006) reported altered maternal nursing behavior and serum prolactin content in the *Clock*-Δ*19* line of mice, however these differences do not likely account for decreased lactation competency, as frequency of nursing bouts increased and there was no difference in basal prolactin levels. Studies in our lab showed that alveolar differentiation in mammary glands from late pregnant *Clock*-Δ*19* mice was impaired compared to WT. Thus, suggesting that circadian clocks affect lactation competency in part through regulation of mammary development (Casey, T.; unpublished results). Moreover, this idea of mammary clock driving mammary gland development is supported also by photoperiod effects on mammary function, as exposure to short day photoperiod (SDPP; 8 h light:16 h dark) vs. long day photoperiod (LDPP) during the dry period increases milk production in the subsequent lactation, in part, by increasing mammary cell proliferation (Mabjeesh et al., 2007; Dahl, 2008).

There is also evidence that the mammary clock regulates expression of genes that regulate milk synthesis. Analysis of effect of decreased levels of CLOCK on HC11 differentiation found that after 96 h of culture with prolactin, glucocorticoids and insulin to induce differentiation, shCLOCK transfected cells expressed significantly lower levels of fatty acid synthase (FASN) and the adherens junction protein CDH1 than wild type HC-11 cells (Casey et al., in revision). *In vivo* studies revealed BMAL1 and PER2 showed circadian patterns of expression in RNA isolated from milk fat globules of lactating sheep which correlated with circadian changes in expression of acetyl-CoA carboxylase (ACACA) as well as percent milk fat (Schmitt et al., 2014). In mice, mammary expression of LALBA (alpha-lactalbumin), SREBF1 (sterol regulatory-element-binding protein 1) and FASN (fatty acid synthase) genes all showed circadian rhythms during lactation (Casey et al., 2014). Circadian rhythms of lactose synthesis is well characterized and known to be mediated by circadian changes of expression in lactose synthesis enzymes (Kuhn et al., 1980). Together, these findings support that circadian clocks regulate metabolic output (milk synthesis) during lactation.

A growing body of literature supports that mammary circadian clocks have two primary functions in the gland. First, circadian rhythms of expression of genes that regulate proliferation and differentiation in combination with dynamic changes in core clock dynamics along the lactation cycle support a role for the mammary clock in regulation of gland development. Secondly, circadian rhythms of genes that regulate milk synthesis, and responsiveness of mammary clock to suckling cues support a role for clock in regulation of synthesis of milk components.

## Connections between serotonin and circadian clocks

As reviewed above, both systems, serotoninergic, and circadian, are present in the brain where they function as principle regulatory networks of homeostatic and homeorhetic processes. These master regulatory systems are extensively intertwined, with neuronal connections, and expression of key genetic elements for serotonin signaling in clock neurons and expression of key clock genes in serotonergic neurons (Ciarleglio et al., 2011). Outside the CNS, circadian and serotoninergic factors regulate key peripheral tissue functions that affect homeostatic and homeorhetic processes, including in the mammary gland.

**Figure 4:**
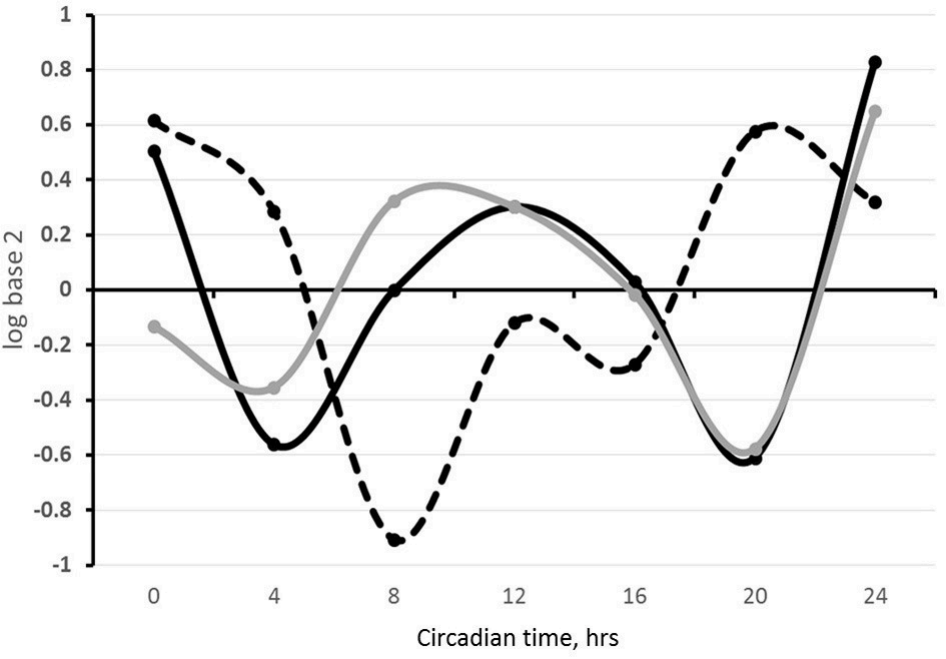
**SLC6A4, BMAL1, and PER2 expression in sheep milk fat globules in a 24 h period**. Total RNA was isolated from milk fat globules collected from lactating sheep every 4 h during a 24 h period the first week postpartum and used for temporal analysis of gene expression. Relative gene expression is expressed as the log base 2 of the 2^−ΔΔCt^ calculations of the gene expression, using the mean across time points within an animal as the normalizer and 18S as the reference gene. Black, gray and dashed lines represent SLC6A4 (SERT), PER2, and BMAL1 expression, respectively.

**Figure 5:**
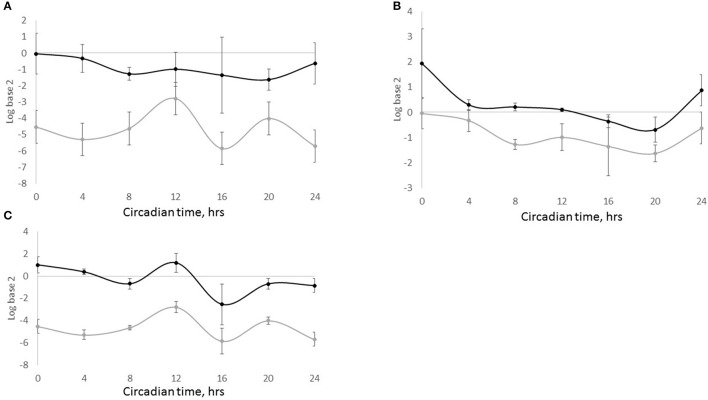
**SLC6A4 and PER2 expression in HC11 and shCLOCK cells**. Cells were plated, grown to confluence and media was changed to synchronize clocks. Samples for mRNA extraction were taken every 4 h over a 24 h period. **(A)** Expression of SLC6A4 in HC11 (black line) and shCLOCK (gray line) cells. **(B)** Expression of PER2 (black line) and SLC6A4 (gray line) in HC11 cells. **(C)** Expression of PER2 (black line) and SLC6A4 (gray line) in shCLOCK cells. Expression of SCL4A4 and PER2 was analyzed by qPCR. Ct results were analyzed using the 2^−ΔΔCt^ method, and CT0 normalization was done.

We propose that similar to central reciprocal regulation of serotoninergic and circadian systems, these systems are connected in the systemic and local regulation of the mammary gland development and lactation performance. Circadian clock dynamics and serotonin components both exhibit dynamic changes in the gland with reproductive-developmental state (Matsuda et al., 2004; Metz et al., 2006; Casey et al., 2014). These changes likely reflect in part their reciprocal regulation, and initial investigations in our laboratory and others support interactions among the circadian and serotonergic systems in the mammary gland. Among these investigations was bioinformatics analysis of the 2000 bp upstream sequences of serotonergic genes: TPH1, SLC6A4, AADC, and HTR7 in mouse genome, which revealed that all four genes have multiple non-canonical E-box sequences (CANNTG). SLC6A4, the gene that encodes SERT, also had a canonical sequence (CACGTG) in this region (Table 1). Temporal analysis of steady state levels of SLC6A4 in total RNA samples isolated from sheep milk fat globules showed the gene exhibits a circadian rhythm of expression. In addition, SLC6A4 pattern of expression was similar to the rhythm founded for PER2 and out-of-phase from BMAL1 expression rhythm (Figure 4). Comparison of PER2 and SERT temporal expression patterns in wild-type HC11 cells, and cultures that carry shRNA which targeted CLOCK, showed SLC6A4 expression was decreased across all the time points in lines with decreased CLOCK abundance relative to controls. Moreover, as with *in vivo* findings, PER2 and SLC6A4 expression patterns were similar in both cell lines, WT and shCLOCK (Figure 5). Thus, suggesting direct, or indirect, regulation of SERT expression by BMAL1-CLOCK. Further, a recent study by Laporta et al. (2015) showed that 5-HT levels corresponded to expression levels of the clock core gene PER2 in mouse mammary glands. This group found a 4-fold increase in mammary PER2 mRNA expression when TPH1 knockout mice were treated with exogenous 5-HTP (5-hydrotriptophan, a serotonin precursor) compared with untreated wild-type mice. Studies in our lab found the addition of 5-HT to mammary explants from lactating mice cultured in prolactin, hydrocortisone and insulin shifted temporal expression patterns of BMAL1, CLOCK, PER1, and PER2 relative to controls cultured in prolactin, hydrocortisone and insulin alone. Together suggesting that 5-HT acts as a direct or indirect input to mammary clocks.

**Table 1:** Upstream location of canonical (CACGTG) and non-canonical^*^ (CANNTG) E-box nucleotide sequences of ***TPH1, SLC6A4, DDC***, and ***HTR7*** transcription start sites.

	***TPH1***	***SLC6A4***	***DDC***	***HTR7***
CACGTG		−1282		
CAAGTG	−874		−71	−943
CAAATG	−1557			
CAACTG	−1051		−1922	−1327
CACATG			−82	
CACTTG	−721 −1924 −1935		−383	−682
CACCTG		−1072 −1436	−1305 −1753	
CAGGTG	−93			−314
CAGATG	−269 −613 −1263			
CAGCTG		−136 −1024 −1720 −1776		−887
CAGTTG	−666			−702
CATATG			−608	−741 −1954
CATCTG		−1706		
CATGTG	−335	−42		−605

* *CAATTG and CATTTG sequences were not present in 2 kb region upstream from analyzed gene start site*.

Another probable way that circadian and serotonergic systems interact to regulate lactation systemically and within the mammary gland is through PRL mediated homeostatic-homeorhetic processes. PRL is a potent lactogen and stimulates homeorhetic processes for adaptation to lactation and photoperiod. PRL regulates serotonergic and circadian systems in mammary, functioning to stimulate 5-HT synthesis and as an important input to mammary clocks. Circulating PRL levels are increased in cattle exposed to long day photoperiod (LDPP, 16 h of light and 8 h of dark). LDPP exposure of pubertal heifers increases mammary parenchyma, while exposure of lactating cows increases milk yield (Dahl et al., 2012). Prolactin is mainly produced by the lactotroph cells in the anterior pituitary, where its secretion is controlled though both prolactin-inhibiting factors, which decrease PRL secretion, and releasing factors (PRFs), which directly or indirectly stimulate PRL secretion from lactotrophs. Nuclei extending from hypothalamus contain dopaminergic cells that tonically inhibit PRL release via projections to the anterior pituitary. Positive releasing factors include oxytocin (OT) and vasoactive-intestinal peptide (VIP). Afferent sources of VIP include the suprachiasmatic nucleus (SCN) and the paravetricular nuclei (PVN). VIP from the SCN is responsible for circadian rhythms of PRL secretion, and suckling induced prolactin release is mediated, at least in part, by suckling induced release of OT. Acute injections of 5-HT or its precursor, 5-HTP, also stimulate PRL release (Kamberi et al., 1971). However, these effects do not appear to be direct, but rather are mediated through VIP and OT. In particular 5-HT neurons innervate both the SCN and PVN to regulate VIP activity and OT secretion (Emiliano and Fudge, 2004). Changes in photoperiod affect relative levels of serotonin-melatonin (Tan et al., 2014). Thus, we propose that in a LDPP, increased serotonin increases PRL secretion through the VIP-OT pathways. PRL in turn binds to cell surface receptors including in the mammary where it influences phase and amplitude of BMAL1 expression.

Prolactin is also synthesized in MECs where it acts in an autocrine manner. Induction of the PI3K-Akt pathway stimulates PRL production in MECs, and autocrine PRL is required for the initiation of lactation (Chen et al., 2012). Serotonin activates the PI3K-AKT pathway in prostate cells via 5-HTR1A (Dizeyi et al., 2011) and cardiomyocytes following binding to 5-HTR2B receptor (Nebigil et al., 2003). MEC expresses 5-HTR2B (Hernandez et al., 2009; Pai and Horseman, 2011), thus it is probable that serotonin activates PI3K-Akt pathway in the mammary gland, which in turn stimulates autocrine prolactin production that can act on the mammary clock. Studies will need to be done to determine if these serotonergic-prolactin-clock interactions occur in an autocrine manner in the mammary gland.

In conclusion, both the circadian and serotonergic systems regulate homeostatic and homeorhetic processes along the lactation cycle. Preliminary evidence support that similar to central mechanisms, circadian clocks and peripheral serotoninergic system interact to mediate homeorhetic and homeostatic processes that support lactation. Each system drives developmental changes in the gland as the female transitions through the lactation cycle, and both systems function as homeorhetic regulators to support changes in metabolic demands with changes in reproductive state (pregnancy-lactation-weaning). Further studies are needed to elucidate mechanism of interactions among the systems in the mammary gland and the role of these interactions in the regulation of the lactation cycle.

## Author contributions

All authors listed, have made substantial, direct and intellectual contribution to the work, and approved it for publication.

## Funding

This work has been supported by FPU 12/06079 Scholarship, and by the scholarship EST 14/00493 both from the Ministry of Education, Culture and Sports of the Spanish Government (Madrid, Spain).

### Conflict of interest statement

The authors declare that the research was conducted in the absence of any commercial or financial relationships that could be construed as a potential conflict of interest.
